# Grading of Frequency Spectral Centroid Across Resting-State Networks

**DOI:** 10.3389/fnhum.2018.00436

**Published:** 2018-10-26

**Authors:** Anja Ries, Catie Chang, Sarah Glim, Chun Meng, Christian Sorg, Afra Wohlschläger

**Affiliations:** ^1^Department of Neuroradiology, Technical University of Munich, Munich, Germany; ^2^TUM-NIC, Neuroimaging Center, Technical University of Munich, Munich, Germany; ^3^Advanced MRI Section, Laboratory of Functional and Molecular Imaging, National Institute of Neurological Disorders and Stroke – National Institutes of Health, Bethesda, MD, United States; ^4^Graduate School of Systemic Neurosciences, Ludwig-Maximilians-Universität München, Munich, Germany; ^5^Department of Psychiatry, University of Cambridge, Cambridge, United Kingdom; ^6^Department of Psychiatry, Technical University of Munich, Munich, Germany

**Keywords:** resting-state, fMRI, resting-state networks, spectral centroid, frequency-based grading, major depressive disorder, spectral analysis, resting-state functional connectivity

## Abstract

Ongoing, slowly fluctuating brain activity is organized in resting-state networks (RSNs) of spatially coherent fluctuations. Beyond spatial coherence, RSN activity is governed in a frequency-specific manner. The more detailed architecture of frequency spectra across RSNs is, however, poorly understood. Here we propose a novel measure–the Spectral Centroid (SC)–which represents the center of gravity of the full power spectrum of RSN signal fluctuations. We examine whether spectral underpinnings of network fluctuations are distinct across RSNs. We hypothesize that spectral content differs across networks in a consistent way, thus, the aggregate representation–SC–systematically differs across RSNs. We therefore test for a significant grading (i.e., ordering) of SC across RSNs in healthy subjects. Moreover, we hypothesize that such grading is biologically significant by demonstrating its RSN-specific change through brain disease, namely major depressive disorder. Our results yield a highly organized grading of SC across RSNs in 820 healthy subjects. This ordering was largely replicated in an independent dataset of 25 healthy subjects, pointing toward the validity and consistency of found SC grading across RSNs. Furthermore, we demonstrated the biological relevance of SC grading, as the SC of the salience network–a RSN well known to be implicated in depression–was specifically increased in patients compared to healthy controls. In summary, results provide evidence for a distinct grading of spectra across RSNs, which is sensitive to major depression.

## Introduction

The human brain is capable of complex functions which are supported by its distinct spatiotemporal organization. Human cortical regions are arranged in a characteristic sensorimotor-to-transmodal gradient, revealing gradual variation in structural features (Huntenburg et al., 2017), macroscopic connectivity (Margulies et al., 2016) and functional specialization (Huth et al., 2016). This spatial gradient is anchored in early sensory cortical areas such as the primary visual, somatomotor, and auditory cortices and evolves toward higher-order transmodal areas in the parietal, temporal, and prefrontal cortex (for review see Huntenburg et al., 2018).

The human brain also exhibits a hierarchy of timescales of neural dynamics. Prior work revealed that during various task demands information integration across spatially distinct neural circuits evolves on different timescales (Hasson et al., 2008, 2015; Lerner et al., 2011; Ding et al., 2016; Baldassano et al., 2017). Primary sensory areas were found to encode instantaneous, rapidly changing information whereas transmodal association areas were shown to encode information accumulated over a longer time. Such temporal hierarchy of information integration during task relates to timescales of intrinsic (resting-state) cortical dynamics (Honey et al., 2012; Stephens et al., 2013; Murray et al., 2014). Correspondingly, early sensory areas that accumulate information over shorter timescales were found to exhibit faster resting-state fluctuations while transmodal areas that accumulate information over longer timescales showed slower resting-state fluctuations. Numerous other studies in humans and primates further support the notion of a hierarchy of resting-state timescales across individual cortical regions (Baria et al., 2011; Chaudhuri et al., 2015; Cocchi et al., 2016).

Intrinsic, slowly fluctuating brain activity is organized in so called resting-state networks (RSNs) of coherent activity (Biswal et al., 1995; Greicius et al., 2003; Fox et al., 2005; Fox and Raichle, 2007). Analysis of RSN activity and their interactions–typically by correlated resting-state fMRI signal fluctuations–constitute a powerful tool to study large-scale functional integration of the brain. Functional connectivity (FC) is a measure of signal covariance and allows for the assessment of communication between spatially distinct brain regions within (intra-FC) or between (inter-FC) networks.

Consistently with the spatial organization of resting-state dynamics of individual cortical regions, network activity at rest is also largely shaped by the temporal domain. Both intra- and inter-FC as well as the resulting network topologies–assessed via fMRI blood oxygen level dependent (BOLD) signal–were shown to be governed in a frequency specific manner (Salvador et al., 2008; Wu et al., 2008; Zuo et al., 2010; Sasai et al., 2014; Thompson and Fransson, 2015). More recently, Gollo et al. (2017) suggested that a hierarchy of timescales organizes activity between RSNs (with higher order networks showing a slower regime of activity and sensory networks faster neural dynamics) and within RSNs (with highly connected regions of a network showing slower dynamics than less interconnected, peripheral regions). Considering these findings, it is crucial to investigate brain’s functional organization in a frequency-resolved fashion.

Intact temporal organization of neural dynamics determine a healthy regime of brain functioning. Deviations from this healthy regime could be reflected in malfunctioning neural processes, as is the case in several neurological disorders. Alterations in power spectra of resting-state fluctuations in individual brain regions or networks have been reported in schizophrenia (Garrity et al., 2007; Calhoun et al., 2011), bipolar disorder (Calhoun et al., 2011), and chronic pain (Malinen et al., 2010; Baliki et al., 2011). Moreover, there are numerous reports of altered FC in neurological disorders such as schizophrenia (Garrity et al., 2007; Rotarska-Jagiela et al., 2010; Dong et al., 2018), Alzheimer’s disease (Greicius et al., 2004; Sorg et al., 2007; Wang et al., 2007; Sheline and Raichle, 2013; Badhwar et al., 2017), obsessive-compulsive disorder (OCD) (Zhu et al., 2016; Gürsel et al., 2018), and major depressive disorder (MDD) (Greicius et al., 2007; Wang et al., 2012; Kaiser et al., 2015; Zhong et al., 2016). Aberrant FC patterns could relate to shifts in frequency distribution of regional and network BOLD signal fluctuations. Nonetheless, spectral properties of BOLD fluctuations in many of these disorders have not yet been investigated or need to be investigated in more detail to better understand the underlying causes of the breakdown in large-scale brain communication.

As such, well-functioning interregional FC is viewed as a prerequisite of large-scale communication of neuronal assemblies, where the ongoing oscillatory processes of individual brain areas as well as of widespread networks constitute the groundwork of FC formation and should be investigated in more detail. Contrary to the initial view on fluctuating properties of RSNs–which narrows the investigatory interest to peak frequencies below 0.1 Hz (Cordes et al., 2001; Fox and Raichle, 2007)–it has been shown that the frequency spectrum of intrinsic fluctuations is broad and includes frequencies up to 0.25 Hz or even 0.75 Hz (Niazy et al., 2011; Boubela et al., 2013; Gohel and Biswal, 2015). Thus, RSNs represent complex processes that evolve through coherence on various temporal scales within the broad term of low-frequency fluctuations. Investigation of broadband processes is needed to preserve the richness of RSNs’ operating regime, i.e., information content across the broad frequency spectrum. The more detailed architecture of frequency spectra across networks is, however, poorly understood.

Identifying key features of broadband spectra of BOLD network fluctuations–which might be also sensitive to pathological change–however, bears a challenge, given the relatively wide frequency span of meaningful resting-state BOLD fluctuations (0.01–0.75 Hz) and the need for frequency-resolved analysis (for example dividing the full frequency band into 3–10 sub-bands). Moreover, the vast amount of literature on aberrant resting-state BOLD activity in various diseases–often involving divergent results–points toward a vast number of regions or networks that are implicated in specific diseases. Thus, investigating group differences in spectral properties of regions/networks-of-interest within several frequency sub-bands would involve many statistical tests, possibly inflating false-positive results; and if strictly corrected for multiple comparisons–reduce the statistical power of the analysis. To circumvent this problem, complex measures can be summarized into meaningful aggregate measures, which may improve detection of systematic patterns and emphasize major disease-related alterations. These subsequently can be followed up by *post hoc* tests and targeted investigations yielding detailed information on the underlying changes in power spectra.

To validate the usefulness of the proposed approach, we investigate activity fluctuations within RSNs with special focus on the broad range of dominant frequencies involved in their time courses–assessed via an aggregate measure of BOLD spectral properties within a network. To carry out such analysis, we propose a novel measure for RSN activity analysis–the Spectral Centroid (SC). The SC of BOLD network fluctuations represents the “center of gravity” of the full power spectrum. Pictorially, it can be understood as a midpoint within the spectral density function at which the distribution is divided into two equal parts so that, figuratively speaking, if put on the tip of a pin at this midpoint–the spectral distribution would be perfectly balanced. In mathematical terms it represents a weighted mean, as explicitly given in the methods section below. In practice, the SC is a compact measure for statistical analysis. The SC has been applied in a recent study–paralelly to our investigations–to examine the slowing of neural dynamics during graded sedation with propofol (Huang et al., 2018; in this study, the SC is referred to as the “mean frequency”).

We hypothesize that each RSN exhibits a distinct SC value, therefore, each RSN takes a distinct position in an ordering of all SC values–which is referred to as grading of SC across RSNs. We also aim to explore whether the grading of SCs across networks fits the prior observations of sensorimotor-to-transmodal gradient of neural timescales. Furthermore, we hypothesize that a given brain disease would alter specifically such graded organization across networks. To test our hypothesis of distinct SC value in healthy subjects, we calculate the SC using high-quality and large sample size (*N* = 820) rs-fMRI data of the Human Connectome Project (HCP^1^) (Smith et al., 2013; Van Essen et al., 2013). To validate the reliability and replicability of results on SC obtained from the HCP dataset–along with general concerns about replicability in neuroimaging studies (Poldrack et al., 2017)–we further examine the SC based on an independent dataset of 25 healthy subjects, acquired at our research facility. Furthermore, to examine the potential biological relevance of SC we investigate the SC grading in a major brain disease, namely MDD, in which impairments in communication (expressed through FC) between and within networks have been widely reported (for review see Kaiser et al., 2015); in particular, the major body of evidence shows abnormalities for MDD in the salience network (SN) (Seeley et al., 2007; Menon and Uddin, 2010), default mode network (DMN) (Raichle et al., 2001; Buckner et al., 2008) and central executive network (CEN) (Seeley et al., 2007; Vincent et al., 2008). Since the frequency content of a network’s signal is crucial to the formation of FC with other networks, we hypothesize that aberrant FC in depression relates to possible shifts in spectral power of infra-slow BOLD fluctuations of these RSNs.

## Materials and Methods

Two separate datasets were used for this work: Dataset 1 comprises rs-fMRI images from 820 healthy subjects of the HCP, Dataset 2 comprises structural MRI and rs-fMRI data of 25 healthy controls (HCs) and 25 MDD patients–acquired at our research facility.

### Dataset 1: Human Connectome Project

#### Data Specification

We calculated the SC on a large sample size dataset of HCP from the 900 Subjects Release (S900) to present the applicability and meaningfulness of the SC as an aggregate measure of underlying oscillatory features of a network. Among other imaging modalities (task-based MEG and fMRI, structural scans), the S900 release contains rs-fMRI scans of healthy subjects, with four rs-fMRI runs collected per subject. Out of 900 subjects, the full acquisition of all four runs with 100% of collected timepoints was completed only in 820 subjects. Our analysis is based on the rs-fMRI data of those 820 subjects. Specifically, we analyze the SC on network time courses of independent components (ICs), which are freely provided for download.

Detailed information about the HCP acquisition protocols are described elsewhere (Smith et al., 2013; Uğurbil et al., 2013; Van Essen et al., 2013^2^). In summary, participants’ functional images in Dataset 1 were acquired in four runs of approximately 15 min each. Two runs were acquired in one session and two in another session. Participants were asked to keep their eyes open with relaxed fixation on a projected bright cross-hair on a dark background, presented in a dimmed room. Within each session, oblique axial acquisition alternated between phase encoding in right-to-left (RL) direction in one run and phase encoding in a left-to-right (LR) direction in the other run. Resting-state images were collected with the following parameters: gradient-echo EPI sequence, TR = 720 ms, TE = 33.1 ms, flip angle = 52°, field of view = 208 × 180 (RO × PE), matrix 104 × 90 (RO × PE), slice thickness = 2 mm, 72 slices, 2.0 mm isotropic voxels, multiband factor = 8, echo spacing 0.58 ms, and bandwitdth = 2290 Hz/Px.

Within the HCP framework, fMRI data were preprocessed using the minimal preprocessing pipeline (MPP) (Glasser et al., 2013). Afterward, rs-fMRI data were denoised using FMRIB’s ICA-based Xnoiseifier (FIX) (Griffanti et al., 2014; Salimi-Khorshidi et al., 2014). FIX is used to auto-classify ICA components into “good” vs. “bad” ICs, and bad components are subsequently removed from the fMRI data.

Next, ICA on preprocessed and denoised (MPP + FIX) data was performed by HCP using FSL MELODIC software^3^. Different ICA parcellation scenarios were used with varying number of ICs (15, 25, 50, 100, and 300). For each scenario, averaged time courses per IC and subjects are publicly available for download^4^.

#### Data Analysis

We based our analysis on the 50 ICs scenario. From the 50 ICs, we identified 24 RSNs in an automated way using *fslcc*–a FSL based function–by calculating cross-correlations (threshold *r* > 0.2) between 50 ICs from Dataset 1 and the established RSN templates from Allen et al. (2011) (freely available for download^5^).

##### Spectral centroid

Power spectra of BOLD network fluctuations were estimated using Welch’s averaged periodogram method. Welch-periodograms were calculated on time courses of each RSN per participant and run–using the built-in Matlab function *pwelch*, with a Hamming window of 50% overlap of successive windows–to obtain power estimates for each spectral bin. With a TR of 720 ms the full spectrum of accessible frequencies spans up to 0.69 Hz (Nyquist frequency). Results of Welch’s method were restricted to the frequency range of 0.01–0.69 Hz, omitting the very low frequencies (<0.01 Hz) as these are largely affected by slow drifts due to scanner hardware and cannot fully be separated from neuronal drifts. Spectral properties of the infra-slow BOLD network fluctuations were examined at the individual level by means of the SC. The SC is an integral measure obtained by evaluating the “center of gravity” of the spectrum based on frequency and magnitude information from the Welch-periodogram. The SC is calculated as the weighted mean of the frequencies present in the signal with their power as the weight, see formula below:

(1) $$SC=\frac{\sum_{i=1}^{N/2+1}i\times f\times P(i)}{\sum_{i=1}^{N/2+1}P(i)}$$

where *f* is the width of each spectral bin in Hz, *P(i)* is the power at the *i*th spectral bin given in Hz, and *N* is the number of points in the network’s BOLD time series. In Dataset 1 the respective parameters are: *f* = 0.0012 Hz, and *N* = 1200.

We calculated the SC of the power spectrum of BOLD network fluctuations–within the frequency range of 0.01–0.69 Hz–for each of the 24 RSNs per participant and run. Thus, we obtain four SC values per subject and per RSN. For each RSN and subject, the mean SC of all four runs was calculated and is later referred to as the SC. In the end, we obtain one mean SC value per subject and per RSN.

In Matlab, Lilliefors tests were carried out on the set of SC values for each RSN to determine whether they follow a normal distribution (*p* < 0.05, Bonferroni-corrected for 24 RSNs). SC values in some of the RSNs were found to deviate from normal distribution, thus, further analysis was performed applying non-parametric tests. The Friedman’s test–a non-parametric alternative to repeated measures ANOVA–was performed on the SC values with RSN as main factor. *Post hoc* tests were performed as Wilcoxon signed rank test for investigating significant pairwise differences in SCs between RSNs in Dataset 1 (*p* < 0.05, Bonferroni-corrected for 24 RSNs).

##### Assessment of SC dependence on RSN size

Additionally, we investigated the relation between RSN size and its corresponding SC value. This was motivated by the possibility that, despite the high quality of provided data and careful artifact removal, spatially smaller RSNs could be differentially affected by local high-frequency motion artifacts compared to more distributed networks. The size of a given RSN was computed from group-averaged IC spatial maps provided by HCP. These spatial maps were first binarized (at a threshold of *z* > 10, which corresponds to *p* < 0.001 with FWE correction), next non-zero voxels were counted, and the sum of these voxels indicated the size of a network. Pearson’s correlation was computed between RSN size and SC value. The results and interpretation of this analysis are presented in Supplementary Material.

### Dataset 2: Healthy Controls and MDD Patients

#### Subjects

This dataset comprised anatomical and functional MRI images from 25 healthy individuals and 25 patients who suffered from recurrent MDD (see Table 1 for detailed information on demographical and clinical characteristics). Participants’ data have also been used in previous studies (Manoliu et al., 2013; Meng et al., 2014). Patients with MDD were recruited from the Department of Psychiatry of the Klinikum rechts der Isar, Technische Universität München by psychiatrists. HC individuals were recruited via advertising. All participants provided informed consent in accordance with the Human Research Committee guidelines of the Klinikum rechts der Isar, Technische Universität München. All participants were examined for their medical history, underwent psychiatric interviews and psychometric assessments. Psychiatric diagnoses were based on Diagnostic and Statistical Manual of Mental Disorder–IV (DSM–IV) (American Psychiatric Association, 2000). The Structured Clinical Interview (SCID) for DSM–IV was used to assess the presence of psychiatric diagnoses (First et al., 1996). The severity of depression symptoms was measured with the Hamilton Rating Scale for Depression (HAM-D) (Hamilton, 1960), as well as the Beck Depression Inventory (BDI) (Beck et al., 1961). The global level of social, occupational, and psychological functioning was measured with the Global Assessment of Functioning Scale (GAF) (Spitzer et al., 1992). The clinical-psychometric assessment was performed by psychiatrists who have been professionally trained for SCID interviews with inter-rater reliability for diagnoses and scores of >95%. Recurrent MDD was the primary diagnosis for all patients. More detailed patient description including disease history, co-morbidity, and current medication may be found in Meng et al. (2014) as well as in the Supplementary Material.

**Table 1:** Demographic and clinical characteristics.

Measure	MDD (*n* = 25)	HC (*n* = 25)	MDD vs. HC^a,b^
	**Mean (*SD*)**	**Mean (*SD*)**	***p*-value**
Age [years]	48.76 (14.38)	44.08 (14.78)	>0.05^a^
Gender (m/f)	12/13	11/14	>0.05^b^
Duration of MDD [years]	16.72 (10.20)	NA	
Number of episodes	5.56 (2.47)	NA	
Duration of current episode [weeks]	16.56 (6.62)	NA	
GAF	49.80 (10.53)	99.50 (1.10)	<0.001^a∗^
HAM-D	22.12 (7.06)	0	<0.001^a∗^
BDI	24.08 (6.31)	0	<0.001^a∗^


*^a^Two-sample t-test. ^b^χ^2^-test. ^∗^p < 0.05, Bonferroni-corrected for multiple comparisons. MDD, major depressive disorder; HC, healthy controls; SD, standard deviation; GAF, Global Assessment of Functioning Scale; HAM-D, Hamilton Depression Rating Scale; BDI, Beck Depression Inventory.*

#### Data Acquisition and Preprocessing

10 min of resting-state functional MRI was acquired from all participants who were instructed to keep their eyes closed, not to think of anything particular, and not to fall asleep. We obtained subjective verification that participants stayed in a state of alertness during the rs-fMRI scan by interrogating them via intercom immediately afterward. The scanning session was successfully completed in all subjects, and all subjects reportedly stayed awake during the scan.

##### Data acquisition

MRI data were collected on a 3-Tesla Philips Achieva scanner with an 8-channel phased-array head coil. T1-weighted structural images were acquired with a MPRAGE sequence (echo time = 4 ms, repetition time = 9 ms, inversion time = 100 ms, flip angle = 5°, field of view = 240 × 240 mm, matrix = 240 × 240, 170 slices, slice thickness = 1 mm, and 0 mm interslice gap, voxel size = 1mm × 1mm × 1 mm). Functional MRI data were obtained by using a gradient echo EPI sequence (echo time = 35 ms, repetition time = 2000 ms, flip angle = 82°, field of view = 220mm × 220 mm, matrix = 80 × 80, 32 slices, slice thickness = 4 mm, and 0 mm interslice gap, voxel size = 2.75 mm × 2.75 mm × 4 mm; 300 volumes).

##### Preprocessing

The first three volumes of rs-fMRI data were discarded due to magnetization effects. The remaining data were preprocessed with statistical parametric mapping software SPM12^6^, including head motion correction, spatial normalization into the Montreal Neurological Institute (MNI) standard space, and spatial smoothing with a 6-mm full width at half maximum (FWHM) Gaussian filter. No slice-timing correction was performed as prior studies have shown minimal effects of slice-timing correction on fMRI data acquired at a TR of 2s (Wu et al., 2011). Several parameters relating to head motion were investigated and compared between patients and healthy individuals to control for potential differences in motion between groups and potential bias on the results. This included the estimation of temporal signal-to-noise ratio and point-to-point head motion for each subject (Murphy et al., 2007; Van Dijk et al., 2012). Excessive head motion (cumulative motion translation or rotation >3 mm or 3° and mean point-to-point translation or rotation >0.15 mm or 0.1°) was applied as an exclusion criterion. Point-to-point motion was defined as the absolute displacement of each brain volume compared with its previous volume. None of the participants had to be excluded. Moreover, two-sample *t*-tests showed no significant differences between groups regarding mean point-to-point translation or rotation of any direction (*p* > 0.1), as well as temporal signal-to-noise ratio (*p* > 0.5).

##### Nuisance covariates regression

When investigating between-group differences in SC of network BOLD fluctuations, we control for the overall percent signal change (PSC), which provides information complementary to that of SC and may help to elucidate observed differences in SC. PSC has been shown to differ between different RSNs (van den Heuvel et al., 2016) and might be impacted by disease (Sanacora et al., 2002; Brambilla et al., 2003; Tunnicliff and Malatynska, 2003).

Thus, throughout the analysis, we operate on two branched datasets: (1) ICA-derived time courses of RSNs–used in the examination of networks’ spectral properties; (2) preprocessed whole-brain fMRI time courses–used for calculation of PSC. On that account, it is important to maintain the signal properties as consistent across the two datasets as possible. The data obtained through ICA is largely cleaned from non-neuronal variance sources, including respiratory and cardiac signals, head-movement distortions, as well as signals from white matter (WM) and cerebrospinal fluid (CSF). During ICA procedure, fluctuations of such origin are captured as ICs and are, to a great extent, separated from the remaining components characterized as RSNs.

The pre-processed whole-brain fMRI data is corrected for head movement, yet its variance may still be influenced by other sources of artifacts. To reduce the impact of these sources on the BOLD signal, regression of nuisance covariates including WM and CSF was performed. For the calculation of PSC of BOLD network fluctuations, it is highly important to exclude the variance resulting from non-neuronal factors.

Nuisance regression was performed as follows: for each participant, binarized region of interest (ROI) masks of the WM and CSF were created from T1 segmentation compartments (binarized at a threshold of 0.9). Averaged CSF and WM signals were extracted separately from individual participant data and served as covariable signals. Nuisance covariates regression was performed on the previously realigned and normalized, but not smoothed data using REST toolbox v1.8^7^. Subsequently, data was spatially smoothed in SPM12 using a 6-mm FWHM Gaussian kernel.

##### Estimation of cardiac and respiratory rates

Physiologic Estimation by Temporal ICA (PESTICA^8^) (Beall and Lowe, 2007) was applied on raw fMRI data to detect the pulse and breathing cycles in individual subjects. In Matlab, fast Fourier transform (FFT) was used to calculate peak frequencies of cardiac and respiratory rate time courses obtained from PESTICA (with a temporal resolution of TR/slice number = 2s/32). Concretely, a Gaussian fit in a search window was applied which corresponded to expectation values for the physiological rhythms (cardiac 55–70 bpm, beats per minute; respiratory 10–24 bpm). Visual check of fit quality was performed. Group differences in cardiac and respiratory rates were tested with two-sample *t*-tests. The tests yielded no significant difference between the groups regarding the cardiac rate (*p* > 0.5), as well as the respiratory rate (*p* > 0.5).

#### Data Analysis

##### Determination of resting-state networks

Pursuing the established approach from Allen et al. (2011), preprocessed whole-brain fMRI data were decomposed into 75 spatial ICs within a group-ICA framework (Calhoun et al., 2001) based on the infomax-algorithm and implemented in the GIFT software^9^. Data were concatenated and reduced by two-step principal component analysis (PCA). Next, 75 ICs were estimated by use of the Infomax algorithm, which was repeated 20 times using ICASSO^10^ to reach an estimate of component reliability. The resulting set of average group ICs was then back projected into single subject space via GICA. Each back-reconstructed component is described by a spatial map of *z*-scores reflecting the FC pattern of the component across space, and an associated time course reflecting the component’s BOLD activity across time. Voxels exhibiting high FC within the component have high *z*-scores and voxels that fall outside of the networks FC pattern have *z*-scores of approximately 0. A threshold of 1 was set on the *z*-values to omit negative FC patterns within a network. The variance of component’s time course after back projection is by default normalized to 1. For an automated identification of networks-of-interest, multiple spatial regression analyses on the 75 ICs were applied using established and online available templates from Allen et al. (2011) reflecting canonical RSNs. Components of highest correlation coefficient with the templates (threshold *r* > 0.2) were selected for further analysis, resulting in 24 RSNs of interest.

##### Percent signal change

The spectral properties of fMRI time series would be impacted by the relative balance of signal (BOLD contrast) and noise present in the time series. The spectrum of BOLD fluctuations at rest has an approximately 1/*f*^β^ distribution with frequency *f* and power-law exponent β (He, 2011), due in part to the low-pass character of the hemodynamic response. Therefore, assuming that low-frequency noise sources (e.g., aliased physiological noise, slow head motion, and instrumental drifts) are adequately removed, areas with smaller BOLD signal fluctuations may exhibit a distribution skewed toward higher frequencies (i.e., a shallower 1/*f* slope) and reduced overall variance. When examining between-group differences in SC, it is important to control for the influence of PSC on the SC, as there might be systematic differences in PSC related to pathology. Therefore, we also examined the impact of controlling for fMRI signal variance (i.e., PSC) in the analysis of SC.

To calculate the PSC, we first extracted the averaged time course corresponding to a given RSN in each subject from the preprocessed whole-brain fMRI data with and without nuisance regression. For that, binary masks for each RSN and each participant were created individually with a threshold of *z* > 2.32, which corresponds to a *p*-value of 0.01. We obtained two time courses for each network and participant (here called network_EPI_nuisance and network_EPI, respectively, for preprocessed fMRI time courses with and without nuisance regression), the PSC was calculated using the standard deviation (SD) of gray matter based signal (network_EPI_nuisance) and the mean of network_EPI signal, adhering to the established standard procedures for task-based fMRI (see e.g., Gläscher, 2009):

(2) $$PSC=\frac{SD\;(network\_EPI\_nuisance)}{mean\;(network\_EPI)}\times 100$$

In Matlab, Lilliefors tests were carried out on the set of PSC values for each RSN in each group to determine whether they follow a normal distribution (*p* < 0.05, Bonferroni-corrected for 24 RSNs). No significant deviations from normal distribution were found. Differences in PSC between individual networks and between groups were examined via a repeated-measures ANOVA with main factors RSN and group. *Post hoc* tests were performed as Wilcoxon ranksum test (*p* < 0.05).

##### Spectral density

Power spectra of the BOLD time series from each RSN and each participant were computed in Matlab using Welch’s averaged periodogram method (analogous to the procedure in Dataset 1). These spectra were subsequently used for the calculation of the SCs.

For direct comparisons of spectral power between groups, the full power spectra were then split into 10 frequency bands (freq1: 0.01–0.025; freq2: 0.025–0.05; freq3: 0.05–0.075; freq4: 0.075–0.1; freq5: 0.1–0.125; freq6: 0.125–0.15; freq7: 0.15–0.175; freq8: 0.175–0.2; freq9: 0.2–0.225; and freq10: 0.225–0.25 Hz). The choice of frequency bands was exploratory; it constitutes a compromise between averaging for better power and sufficient spectral resolution. Mean power at each frequency band was computed and rescaled by multiplication with the corresponding PSC value. Wilcoxon ranksum tests were performed on the power values at each frequency band to test for differences between MDD and HC groups within the original as well as the rescaled power spectrum of a given network.

##### Spectral centroid

Spectral properties of the infra-slow BOLD network fluctuations were examined at the individual level by means of the SC (as described in eq. 1, with the following parameters: width of each spectral bin *f* = 0.0017 Hz; number of points in the network’s BOLD time series *N* = 300). Next, results of Welch-periodograms were restricted to a frequency range of 0.01–0.25 Hz, omitting the very low frequencies <0.01 Hz. From these results, we calculated the SC of the power spectrum of BOLD network fluctuations for each participant and each of the 24 RSNs. Thus, we obtain one SC value per subject and per RSN.

In Matlab, Lilliefors tests were carried out on the set of SC values for each RSN in each group to determine whether they follow a normal distribution (*p* < 0.05, Bonferroni-corrected for 24 RSNs). No significant deviations from normal distribution were found. Repeated-measures ANOVA with main factors RSN and group was performed on SC values. *Post hoc* tests were performed as Wilcoxon ranksum tests (*p* < 0.05).

##### Correction of spectral centroids with PSC (SCcorr)

To rule out any differences in spectral properties related to the absolute PSC, we performed a regression analysis on the SC values and the PSC values. Separately in each group, a global regression was performed, i.e., the SC values of all RSNs in all participants within the group were pooled together and regressed against the corresponding PSC values. Regression residuals represented the “new” SC value corrected for PSC (later referred to as corrected SC; SCcorr). Repeated-measures ANOVA with RSN and group as main factors was performed on the SCcorr values.

*Post hoc* tests were performed as Wilcoxon ranksum tests (*p* < 0.05) to test for group differences in SCcorr of specific RSNs, and as Wilcoxon signed rank tests for investigating significant pairwise differences in SCcorr between RSNs in the control group (*p* < 0.05, Bonferroni-corrected for 24 RSNs).

##### Assessment of SC dependence on RSN size

As in Dataset 1, we investigated the relation between RSN size and its corresponding SC as well as SCcorr value. The size of a given RSN was computed from group-averaged binarized RSN spatial maps of HCs (obtained in an earlier step for PSC calculation). Non-zero voxels within each RSN spatial map were counted and indicated the network’s size. No significant correlation between RSN size and SC or SCcorr was found (Pearson’s correlation SC: *r* = -0.119, *p* = 0.58; SCcorr: *r* = -0.197, and *p* = 0.36).

##### Assesment of SC dependence on clinical scores

Pearson’s correlation was calculated between SCcorr values of each RSN and measures of symptom severity (*p* < 0.05, Bonferroni-corrected for 24 multiple comparisons as 24 RSNs were investigated).

### Network SC Correspondence Across Datasets

Correlation analysis was run between SC values of corresponding networks obtained from Dataset 1 and networks from HCs in Dataset 2 [for a clear overview of network correspondence across study sites, i.e., Allen et al. (2011), our study, and the HCP, we refer to Supplementary Table 1]. 22 RSNs from Dataset 1 and Dataset 2 could be matched as representing the same network from Allen et al. (2011) templates. SC depends on the overall measurement length as well as on the sampling frequency, as these two parameters determine which part of the signal spectrum is accessible to analysis. E.g., with a higher sampling rate, higher frequencies can be measured which shifts SC to slightly higher values depending on the power within these additional frequencies. Hence, SC may vary between data acquired at different facilities with different acquisition parameters. To enable the comparison of network SC values between the two datasets, results of the Welch-periodograms in Dataset 1 were restricted to the frequency range of 0.01–0.25 Hz (as this is the frequency range accessible from Dataset 2) and–solely for comparison purposes–new SC per network was calculated on this range, so that it corresponds to the frequency range from Dataset 2. The Basal Ganglia network was excluded from the correlation analysis, as in Dataset 1 its SC exceeded two SDs. This resulted in a correlation analysis between SC values of 21 RSNs from Dataset 1 and 21 RSNs from Dataset 2.

## Results

### Dataset 1: Human Connectome Project

**FIGURE 1:**
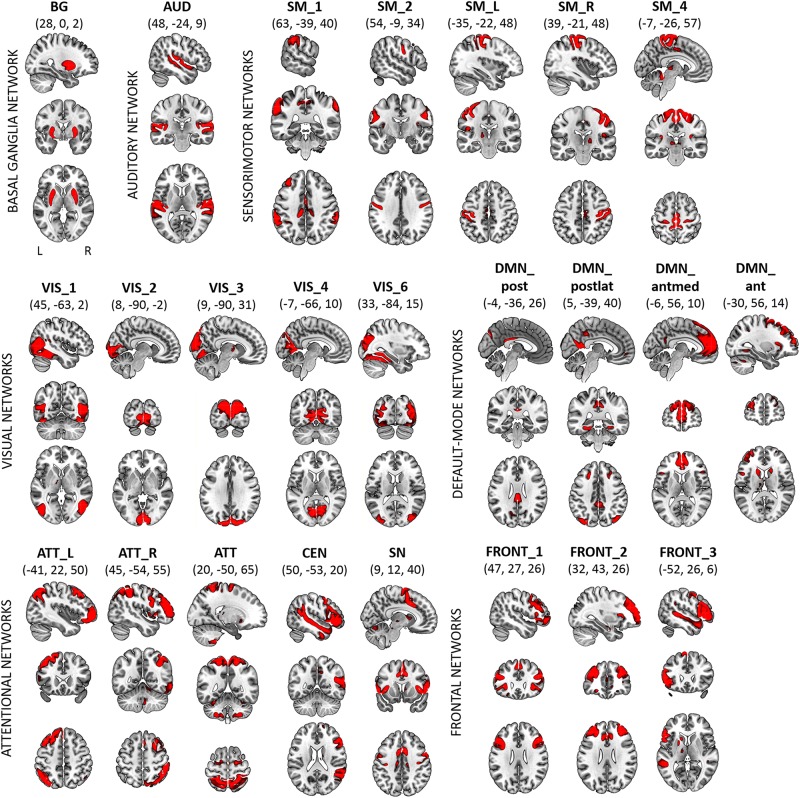
Dataset 1: Spatial maps (SMs) of the 24 independent components (ICs) identified as RSNs, obtained from spatial group-ICA performed by Human Connectome Project. SMs are plotted as *z*-scores and displayed at the three most informative slices (MNI-space). RSNs are categorized into groups according to their anatomical and functional properties. Spatial maps of RSNs were visualized with the mricrogl software (https://www.nitrc.org/projects/mricrogl).

The SC represents the center of gravity of the broad power spectrum of BOLD network fluctuations. We calculated the SC of 24 RSNs that corresponded to networks previously reported by Allen et al. (2011). The selected components from Dataset 1 were classified into the basal ganglia (BG; *n* = 1), auditory (AUD; *n* = 1), sensorimotor (SM; *n* = 5), visual (VIS; *n* = 5), default-mode (DMN; *n* = 4), attentional (ATT; *n* = 5), and frontal (FRONT; *n* = 3) systems. Within the attentional systems, the SN and the CEN were identified. Within the DMN, four subsystems were categorized: the anterior DMN (DMN_ant), anterior-medial DMN (DMN_antmed), posterior DMN (DMN_post) and posterior-lateral DMN (DMN_postlat). The correspondence between canonical RSN templates from Allen et al. (2011) and networks identified from Dataset 1 is described in Supplementary Table 1. Spatial maps of the 24 RSNs from Dataset 1 are shown in Figure 1. Peak activations of RSNs from Dataset 1 are summarized in Supplementary Table 2.

**FIGURE 2:**
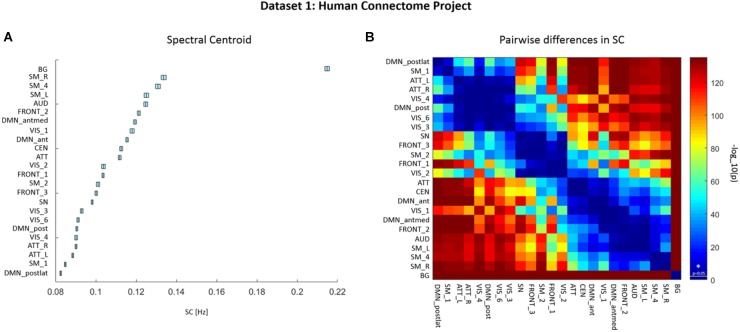
Dataset 1: **(A)** Mean ± SEM spectral centroid (SC) values of each RSN from Human Connectome Project (HCP) data of 820 healthy subjects. RSNs are ordered according to increasing SC magnitude. SCs were calculated based on power spectra of BOLD network fluctuations within the frequency range of 0.01–0.69 Hz. **(B)** Matrix representing *p*-values of pairwise differences in the SC between individual RSNs. The colors indicate the significance, where red stands for lower *p*-value and higher significance, and blue for higher *p*-value thus lower significance. *P*-values were scaled with a –log10(*p*) transform, the colorbar value of 1.3 corresponds to *p* = 0.05 (as indicated by a vertical line across the colorbar and an asterisk which represent significance threshold).

We assessed the SC values from ICA-derived time courses (with a spectral range of 0.01–0.69 Hz) of each RSN and each participant from the HCP data. Mean SC values for each RSN are depicted in Figure 2A and summarized in Supplementary Table 2. The Friedman’s test on SC values yielded a significant effect of factor RSN (χ^2^(23) = 33554.1, *p* < 0.001). Figure 2B displays a matrix of significant pairwise differences in SC between RSNs. Columns and rows, respectively, are ordered according to SC value.

Significant grading of SC values across RSNs is observed in HCP data, implicating that SC values are characteristic for given networks, and most of the networks can be distinguished based on their SC.

### Dataset 2: Healthy Controls and MDD Patients

#### Resting-State Networks

Via ICA, we identified 24 RSNs that corresponded to networks previously reported by Allen et al. (2011). The selected components were classified into the basal ganglia (BG; *n* = 1), auditory (AUD; *n* = 1), sensorimotor (SM; *n* = 6), visual (VIS; *n* = 5), default-mode (DMN; *n* = 4), attentional (ATT; *n* = 5), and frontal (FRONT; *n* = 2) systems. Within the attentional systems, the SN and the CEN were identified. Spatial maps of the 24 RSNs of Dataset 2 are shown in Supplementary Figure 1 and their peak activation sites are summarized in Supplementary Table 3. Correspondence between network templates from Allen et al. (2011) and names of RSNs identified from our data, as well as from HCP data is shown in Supplementary Table 1.

#### Spectral Centroid

**FIGURE 3:**
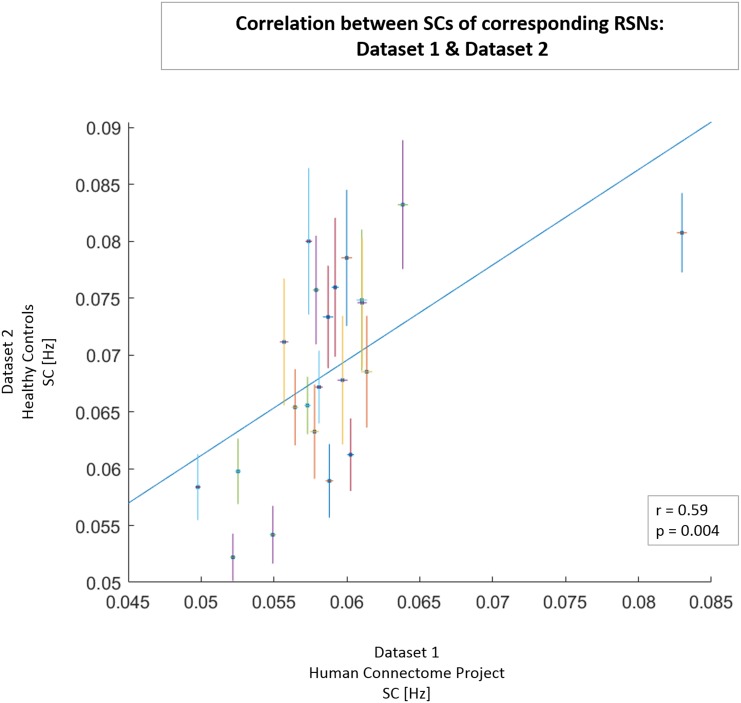
Correlation between SC values of 21 networks obtained from Dataset 1 from signal in frequency range of 0.01–0.25 Hz (*x*-axis) and the corresponding networks obtained from healthy controls in Dataset 2 (*y*-axis). For each network, the horizontal line represents the SEM in Dataset 1, and the vertical line represents the SEM in Dataset 2. A least-squares fit line was added to scatter plot. In the lower right box, correlation coefficient *r* and *p*-value are presented.

We calculated the SC value for each of the 24 RSNs in HC and MDD patients (Dataset 2). The SC values of RSNs from healthy individuals in Dataset 2 and the corresponding SC values of RSNs in Dataset 1 (HCP data; obtained from power spectra restricted to 0.01–0.25 Hz for comparison purposes) showed a significant positive correlation (*r* = 0.59, *p* = 0.004; see Figure 3). From Figure 3 it is also clearly visible that networks of Dataset 1 exhibit smaller within-network variability in SC values (as measured with the standard error of the mean) than networks of HCs from Dataset 2.

**FIGURE 4:**
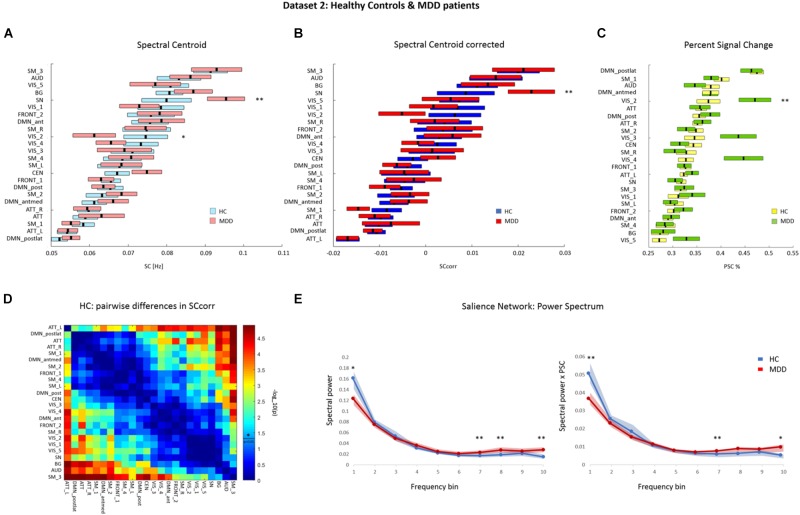
Dataset 2: **(A)** Mean ± SEM SC values of each RSN in HCs and MDD patients. RSNs are ordered according to increasing SC magnitude in HC. SCs were calculated based on the power spectra of BOLD network fluctuations within the frequency range of 0.01–0.25 Hz. **(B)** Mean ± SEM SC values corrected for percent signal change (PSC) in HC and MDD, ordered according to increasing corrected SC magnitude in HC. **(C)** Mean ± SEM PSC values of each RSN in HC and MDD patients. RSNs are ordered according to increasing PSC magnitude in HC. Ordering on the y-axis differs between plots. **(D)** Matrix representing *p*-values of pairwise differences in the corrected SC between individual RSNs in HC. The colors indicate the significance, where red stands for lower *p*-value and higher significance, and blue for higher *p*-value thus lower significance. *P*-values were scaled with a –log10(p) transform, the colorbar value of 1.3 corresponds to *p* = 0.05 (as indicated by a vertical line across the colorbar and an asterisk which represent significance threshold). **(E)** Mean and SEM spectral power of SN at 10 frequency bins in HC (blue) and MDD patients (red). Left panel represents the original power spectrum. Right panel represents the power spectrum rescaled by PSC, where spectral power values for each frequency bin and each subject were multiplied by the corresponding PSC value of SN BOLD fluctuations. (^∗∗^) and (^∗^) indicate significant difference and a tendency toward significance, respectively, in a given measure between groups**–**tested with corresponding statistic. ATT, attention; AUD, auditory; BG, basal ganglia; CEN, central executive network; DMN, default-mode network; FRONT, frontal; SM, sensorimotor; SN, salience network; VIS, visual; L, left; R, right; ant, anterior; antmed, anterior-medial; post, posterior; postlat, posterior-lateral.

Repeated-measures ANOVA on the SC values of Dataset 2, assessed from the spectral range of 0.01–0.25 Hz, yielded a significant main effect of factor RSN (*F*(1,23) = 20.64, *p* < 0.001), and a significant interaction between factors RSN and group (*F*(1,23) = 1.58, *p* < 0.05). What drives the interaction can be deduced from Figure 4A, i.e., the bars representing SC values in separate groups are apart for SN and VIS_2. A *post hoc* Wilcoxon ranksum test yielded significant group differences in the SC of the SN (*p* < 0.05; HC mean ± std: 0.079 ± 0.031 Hz, MDD mean ± std: 0.095 ± 0.024 Hz), and a trend toward significance in one of the visual networks (VIS_2; *p* = 0.06; HC mean ± std: 0.075 ± 0.028 Hz, MDD mean ± std: 0.061 ± 0.027 Hz). Mean SC values for each RSN in both groups are depicted in Figure 4A and summarized in Supplementary Table 4. To control for a potential interaction between power spectra and total power, PSC was calculated and regressed out from the SC values.

#### Percent Signal Change

On PSC values, repeated-measures ANOVA yielded a significant main effect of factor RSN (*F*(1,23) = 11.5, *p* < 0.001) as well as a significant interaction between factors RSN and group (*F*(1,23) = 2.74, *p* < 0.001). Wilcoxon ranksum test was performed as part of *post hoc* analysis to define which RSNs show significant alterations in PSC between the groups. This analysis yielded a significant group difference in PSC in one of the visual networks (VIS_2; *p* < 0.05). Within this network, increased PSC was observed in MDD patients (mean ± std: 0.471 ± 0.162%) compared to controls (mean ± std: 0.374 ± 0.119%). The PSC in another visual network (VIS_4) showed a trend toward significance (*p* = 0.065), with higher values in MDD patients (mean ± std: 0.447 ± 0.198%) than in controls (mean ± std: 0,327 ± 0.078%). Mean PSC values for each RSN in both groups are depicted in Figure 4C and summarized in Supplementary Table 4.

#### Corrected Spectral Centroid

The original SC values were corrected for differences in PSC at the level of each RSN and participant. A repeated-measures ANOVA was performed on the corrected SC (SCcorr) values with RSN and group as main factor. This analysis yielded a significant main effect of factor RSN (*F*(1,23) = 17.84, *p* < 0.001). No significant interaction between factors RSN and group was observed (*F*(1,23) = 1.14, *p* > 0.05). The averaged SCcorr values for each RSN, separately for controls and MDD patients, are displayed in Figure 4B and summarized in Supplementary Table 4.

Figure 4D displays a matrix of significant pairwise differences in SCcorr between RSNs in HCs. Columns and rows, respectively, are ordered according to SCcorr magnitude. The results of Wilcoxon signed rank tests on the SCcorr values between each pair of RSNs in HCs showed significant pairwise differences in all networks with a minimum difference in SCcorr of 0.0001. At the given statistical power, only differences between networks from both ends of the SCcorr scale become statistically significant.

Since the analysis of SC before the correction for PSC showed significant differences in the SC of the SN, and because we hypothesized a disturbance in the oscillatory pattern of the SN specifically–as it is widely implicated in depression–a *post hoc* analysis was performed to investigate group differences in SCcorr values of the SN. Moreover, since we observed a trend toward significance in the SC of visual network VIS_2, in the *post hoc* analysis we also investigated whether the SCcorr of this network significantly differs between groups.

The Wilcoxon rank-sum test revealed a significant difference in the SCcorr of the SN between groups (*p* < 0.05), where MDD patients showed higher SCcorr value (mean ± std: 0.023 ± 0.025) than controls (mean ± std: 0.009 ± 0.03). After correction for PSC, there was no more significant group difference in the SCcorr of the visual network VIS_2 (*p* > 0.05). As such, the previously observed shift in SC of visual network VIS_2 could be fully attributed to differences in PSC of this network.

#### Salience Network: Spectral Differences Between Groups

The SC serves as an aggregate measure which is highly sensitive in detecting key changes in spectral properties of BOLD network fluctuations, assessed uniformly from the broad range of accessible frequencies–i.e., without division into frequency sub-bands. As such, the SC initially indicates network-specific spectral alterations, whereafter targeted *post hoc* tests examining power changes at specific frequency sub-bands can be performed. In that way, the drawback of multiple comparisons can be circumvented–as further detailed investigations of power changes at multiple sub-bands are restricted to networks with significantly changed SC. Thus, the SC measure is highly relevant for identifying key changes in broadband BOLD network fluctuations and for driving targeted *post hoc* analyses.

In Dataset 2, a significant change in SCcorr of the SN was observed in MDD patients, compared to HCs. To further investigate the causes of the shift in SCcorr value within the SN, we examined the spectral power of SN fluctuations at each of the 10 frequency bands in both groups. The original power spectrum of the SN, as well as the power spectrum rescaled by multiplication with PSC are shown in Figure 4E. Analysis of the original power spectrum of the SN yielded a significant increase in power in higher frequency bands (freq7 0.15–0.175 Hz; freq8 0.175–0.2 Hz; and freq10 0.225–0.25 Hz) in MDD patients compared to HCs (*p* < 0.05). There was also a tendency of decreased power in the lowest frequency band (freq1 0.01–0.025 Hz) in MDD patients compared to HCs (*p* = 0.07). Within the rescaled power spectrum of SN, we observed a significant decrease in power of the lowest frequency band (freq1) in MDD patients (*p* < 0.05), and a significant increase in frequency band 7 in MDD patients (*p* < 0.05). Additionally, a tendency of increased power in frequency band 10 was observed in MDD patients (*p* = 0.07). Attenuated power in lower frequency band on the one hand, and increased power in higher frequency bands on the other, result in a shift of the SC in SN toward higher frequencies.

#### SCcorr and Symptoms Severity

Pearson’s correlation was calculated between the SCcorr values of each RSN and the symptom severity scores. We found a significant correlation between BDI scores and the SCcorr of the anterior subdivision of the DMN (DMN_ant: *r* = 0.62, *p* = 0.001) and of the posterior subdivisions of the DMN (DMN_postlat: *r* = 0.65, *p* = 0.0006; DMN_post: *r* = 0.58, *p* = 0.002). There was also a trend toward significance in the correlation between BDI scores and the SCcorr of one of the visual networks (VIS_4: *r* = 0.53, *p* = 0.008). No significant correlation between symptom severity scores and the SCcorr of the SN was observed.

However, an indirect influence of altered SN dynamics on depression severity could be drawn based on findings from a subsequent whole-brain seed-based FC analysis of the SN (for methods and results see Supplementary Material). Reduced FC of the SN toward the anterior DMN and one of the visual networks (VIS_4)–two networks which showed significant correlation between their SCcorr and BDI–was found in MDD patients (Supplementary Figure 2). This observation and its putative consequences are further reviewed in the Discussion part.

## Discussion

Spectral analysis of BOLD fluctuations performed on high-quality large sample dataset of Human Connectome Project–including 820 healthy subjects–revealed a significant grading of SC across networks. This observation could successfully be replicated through an independent dataset of 25 healthy subjects. Network orderings in both datasets under investigation proved to be highly correlated. Thus, the grading of SC proves to be highly reliable across two independent datasets.

The biological relevance of SC as a meaningful representative of networks’ spectral properties was validated via disorder effect. In a dataset of 25 patients suffering from MDD the grading of SC was found to be altered. Specifically, the SC of the SN showed a significant shift toward higher frequencies.

Additionally, a grading of PSC across RSNs was observed in the data of 25 healthy subjects–implicating significantly distinct levels of absolute BOLD activity within specific neuronal ensembles. The grading of PSC was also affected by major depression, where significant increase in PSC of secondary visual-occipital network was found.

### Grading of Spectral Centroid across RSNs

The first striking finding of our study is the occurrence of a highly organized grading of the SCs of BOLD network fluctuations at infra-slow (i.e., <1 Hz) frequencies in healthy subjects. The SC was introduced as a novel measure describing the center of gravity of the full power spectrum of resting-state BOLD network fluctuations. Our investigations on HCP dataset revealed that different RSNs involve and operate on distinct broadband frequency patterns, via which information can selectively be exchanged between targeted systems. To validate the generalizability and replicability of the finding of SC characteristic for RSNs, we repeated the analysis on an independent dataset of 25 HCs acquired at our research facility. In line with main findings on HCP rs-fMRI data, spectral analysis on our dataset also provided a significant grading of the SCs of the BOLD network fluctuations at infra-slow frequencies. Correlation analysis between the SCs of individual RSNs from Dataset 1 and the corresponding RSNs from Dataset 2 confirms the reproducibility of SC as network property, given the different acquisition sites, parameters and analysis pipeline characteristics. The findings on HCs in Dataset 2 further highlight and support the meaningfulness of SC measure as a representative of underlying neuronal features of RSNs, by means of which a general organization–i.e., grading–of neuronal systems according to their spectral properties can be described.

Such grading is–in electrophysiology–believed to be characteristic for a highly interactive system of network oscillators (Buzsáki, 2006) and enables a degree of communication between oscillators, for only networks with overlapping oscillatory profiles are preferentially able to synchronize with each other and form FC. Respectively, any deviation from a network’s healthy oscillation profile would result in a desynchronization between its own signal and the signal of networks it communicates to, thus, in a breakdown of the brain’s large-scale communication structure. Likewise, in BOLD signal–which relates rather to fluctuations in broadband power of electrical oscillation–FC between any two brain areas can only arise with overlapping frequency spectra. Alterations of the spectra are therefore indicative of lack of communication.

Several studies reported that resting-state BOLD fluctuations show frequency-dependent anatomically restricted spatial structure in the human brain (Salvador et al., 2008; Wu et al., 2008; Zuo et al., 2010; Baria et al., 2011; Kalcher et al., 2014). Salvador et al. (2008) examined frequency-dependent FC profiles and found that limbic and temporal regions display highest level of oscillation coherence in high (0.17–0.25 Hz) and middle (0.08–0.17 Hz) frequency bands, while at low frequencies (<0.08 Hz) greatest connectivity is observed in frontal structures. Convergently, Baria et al. (2011) provided evidence of whole-brain organization of BOLD oscillatory activity within the full spectrum of frequencies available from rs-fMRI. They showed that the power of BOLD fluctuations within high-frequency band (0.15–0.20 Hz) is most prominent in the temporal and sub-cortical regions as well as in the insula; whereas power of BOLD oscillation in low-frequency band (0.01–0.05 Hz) was most accentuated in the prefrontal, occipital, and parietal lobes. Kalcher et al. (2014) showed that cortical regions exhibit highest contribution of low-frequency signals (<0.25 Hz), while time courses in subcortical regions as well as the insula are strongly influenced by high-frequency fluctuations (0.25–1.4 Hz). More recently, a hierarchical organization of timescales of intrinsic dynamics has been suggested, highlighting the sensorimotor-to-transmodal temporal gradient (Honey et al., 2012; Stephens et al., 2013; Gollo et al., 2015, 2017; Cocchi et al., 2016; for review see Huntenburg et al., 2018). According to this gradient, early sensory cortical areas such as the primary visual, somatomotor and auditory cortices operate on faster intrinsic dynamics, while the frequency content becomes dominated by lower frequencies as the gradient shifts toward higher-order transmodal areas in the parietal, temporal and prefrontal cortex. Such organization was also observed in context of large-scale brain networks (Gollo et al., 2015, 2017); early sensory RSNs showed faster dynamics than high-order RSNs; and within RSNs, highly interconnected brain regions exhibited a slower dynamic regime than less interconnected peripheral regions.

In partial accordance to the aforementioned findings, our analysis on HCP data revealed the following networks as showing highest SC values, thus, the strongest influence of high-frequency power on the network fluctuations profile: basal ganglia network (main hubs in putamen, caudate nucleus, and pallidum), early sensorimotor networks (SM_R, SM_4, SM_L; main hubs in precentral gyrus and paracentral lobule), auditory network (main hubs in superior temporal gyrus). On the other end of the power distribution, with relatively low SC values, the following networks were situated: posterior-lateral DMN (main hubs in the posterior cingulate cortex (PCC), precuneus, cuneus, angular gyrus, and IPL) and posterior DMN (main hubs in PCC, precuneus, cuneus, and IPL); sensorimotor network (SM_1, main hubs in the supramarginal gyrus and IPL); bilateral attention networks (ATT_L, ATT_R; main hubs in inferior and middle frontal gyri, IPL, and angular gyrus). Some of the visual networks (VIS_4, VIS_6, and VIS_3) were also observed at the lower end of the spectrum, however, these networks represent higher-order systems, which fall toward the end of a modality’s hierarchy of processing complexity.

**FIGURE 5:**
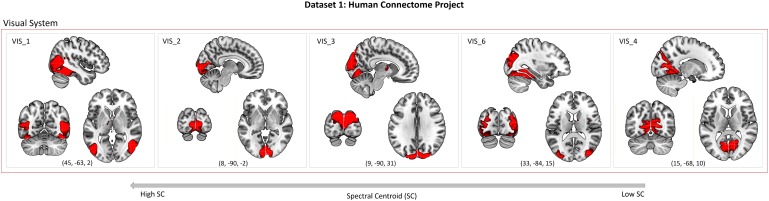
Networks of the visual system ordered accordingly to their decreasing SC magnitude; displayed at the three most informative slices (MNI space).

When ordered accordingly to decreasing SC magnitude, the spatial distribution of brain areas constituting individual visual networks exhibits a rather characteristic pattern (see Figure 5). Networks encompassing predominantly early sensory areas in the striate cortex (primary visual area V1, calcarine sulcus) exhibit a higher SC (VIS_2, VIS_3), thus, their BOLD signal is strongly influenced by high-frequencies. Networks that encompass higher-order cortical areas along the visual stream, i.e., the extrastriate cortex (visual areas V2, V3, V4, and V5/MT) show comparably lower SC (VIS_6, VIS_4), thus, lower-frequencies dominate their BOLD signal. However, there is one exception; VIS_1 shows the highest SC among the visual networks, although it comprises higher-order visual area V5/MT in the middle temporal gyrus. This could be due to the extensive connections between area V5/MT and area V1 (Pascual-Leone and Walsh, 2001; Silvanto et al., 2005), and facilitate direct communication between these two visual areas. Importantly, the finding that networks comprising the same V5/MT area are located both at higher and lower end of the SC gradient could relate to multiple parallel processes being asynchronously undertaken by area V5/MT (for a review see Zeki, 2015). Overall, areas of the visual cortex are highly functionally specialized (Zeki et al., 1991); our results yield that different functional units within the visual system operate on distinct intrinsic dynamics regimes–congruent with the sensorimotor-to-transmodal gradient of timescales.

Interestingly, the subsystems of the DMN (anterior and posterior) were localized at different ends of the power spectrum range, with the anterior and anterior-medial DMN showing relatively higher SC, and the posterior and posterior-lateral DMN showing relatively lower SC. This is indicative of differential contributions of spectral power to different subparts of the DMN, potentially reflecting different processes facilitated via different frequencies. The heterogeneity of the DMN architecture i.e., the subdivision into smaller anatomical-functional subsystems has been shown previously (Andrews-Hanna et al., 2010), and their interplay has been shown to be implicated in the psychopathology of various neuropsychiatric disorders including schizophrenia (Du et al., 2016), Alzheimer’s disease (Damoiseaux et al., 2012), OCD (Beucke et al., 2014) and MDD (Zhu et al., 2012, 2017; Sambataro et al., 2014).

In summary, the grading of SC across RSNs points toward frequency-dependent functional specificity which is indicative of differentiated information integration processes being executed at different frequency scales, in different brain regions. It is in accordance with the results of Neufang et al. (2014) and Cocchi et al. (2016) which hint toward the frequency-driven directionality of information flow in the brain. Differences in spectra of BOLD signals across distinct brain networks have been reported before, via measures of scale-free properties (He et al., 2010; He, 2011). However, measures of scale-free properties alone are not well-suited for explaining findings of network-characteristic processes restricted to limited frequency bands. The SC, on the other hand, both reflects scale-free properties of BOLD signal and carries information about frequency-content–as is it affected by frequency-specific processes.

#### Biological Relevance of Spectral Centroid Grading

Power shifts of BOLD network fluctuations at rest within various frequency bands have been reported in several diseased states. A common tendency of increased power in high frequencies of the BOLD signal and decreased power in low frequencies has been observed in the ACC and insula (Malinen et al., 2010), as well as in the DMN (Baliki et al., 2011) in chronic pain patients; the same tendency was present across many RSNs–including the DMN–in bipolar disorder and schizophrenia (Garrity et al., 2007; Calhoun et al., 2011). The observations of our study are in line with these reports. The grading of SC of BOLD network fluctuations in MDD patients was found to deviate from the healthy orchestration pattern. Specifically, there was a shift of the SC of SN toward higher frequencies, which could be attributed to a relatively decreased power in low frequencies (0.01–0.025 Hz) and increased power in a broad range of higher frequencies (0.15–0.25 Hz) in patients, implying increased activity with characteristic times corresponding to this frequency range within the salience system.

Since the grading of the SC of BOLD network fluctuations is believed to be key to successful communication, we further investigated how shifts in BOLD fluctuations of the SN impact on its communication structure. A subsequent whole-brain seed-based FC analysis of SN enabled us to gain insight into the consequences of its altered dynamic and to specify brain regions toward which SN exhibited decreased FC in patients. In depression, a decreased FC of the SN toward the anterior DMN and one of the visual networks (VIS_4) was observed. Although there was no direct correlation between the SC of SN and depression severity scores, the SCcorr values of the networks to which SN connectivity was disrupted, exhibited a significant correlation with BDI scores. Inasmuch, we suspect an indirect influence that the shift in power spectrum of SN exerts on the severity of experienced symptoms. One possible mechanism underlying such a reaction chain could be that the decoupling of the SN from the anterior DMN and the visual network forces these networks to regain the lost oscillations synchronicity, and that such compensatory attempts would be mirrored through depression severity. We are, however, aware of the speculative character of this interpretation. Further investigations would be needed to support our point of view; this however, is beyond the scope of the current report.

### Grading of Activity Levels Across RSNs

Different RSNs exhibit significantly distinct levels of BOLD activity, as quantified by PSC. Previously, both in the human and macaque cortex, widely distributed brain regions were found to show distinct ratios of excitatory and inhibitory receptor density (ExIn ratio) (van den Heuvel et al., 2016). In humans, regions displaying high ExIn ratios spanned across the precentral, superior frontal, orbitofrontal, supramarginal, superiotemporal, and angular gyri, and the IPL; while regions with relatively low ExIn ratios spanned across the parstriangularis, inferior frontal gyrus and the occipital lobe. Such distribution is to a high extent consistent with the spatial variation in network PSC observed in our study in Dataset 2, where the posterior-lateral DMN (main hubs in superiotemporal, supramarginal, and angular gyri) exhibited by far the highest PSC. It was followed by the sensorimotor network (SM_1; main hubs in the precentral and superior temporal gyri, IPL); the auditory network (main hubs in the superior- and middle temporal, and precentral gyri); and the anterior-medial DMN (main hubs in superior- and middle frontal gyri, and ACC). The lowest PSC was observed in one of the visual networks (VIS_5; main hubs in the lingual, fusiform and middle occipital gyri, and cuneus). We therefore speculate that network PSC is a measure which largely relates to the excitation loading and magnitude of neuronal activity within an ensemble of brain regions, and that the activity profile of a network can be broadly influenced by balanced impact of excitatory and inhibitory neurotransmitters and receptors.

However, in parallel to the coupling between neuronal activity and changes in absolute BOLD signal, it is important to note that differences in BOLD PSC may also be driven by differences in cerebral vascular reactivity (CVR) (Bandettini and Wong, 1997; Handwerker et al., 2007; Thomason et al., 2007), baseline venous oxygenation (Lu et al., 2008), as well as baseline cerebral blood flow (CBF) (Liau and Liu, 2009).

#### Biological Relevance of Activity Level Grading

In addition to our finding of RSNs being characterized by specific PSC values, there was also a deviation in PSC values within networks spanning the occipital cortex in patient group. Specifically, MDD patients showed significantly increased PSC in one of the visual networks (VIS_2; main hubs in the lingual gyrus, cuneus, fusiform gyrus, and occipital gyrus). Our observation of increased PSC in patients’ visual networks can be interpreted in the context of Sanacora et al.’s (2004) reports where, relative to HCs, MDD patients showed altered excitatory and inhibitory neurotransmitter levels in the occipital cortex. Precisely, patients exhibited reduced GABA and increased glutamate concentrations in the occipital cortex, along with decreased GABA/glutamate ratio. These findings, alongside with previous reports of MDD being associated with GABA dysfunction (Sanacora et al., 2000; Brambilla et al., 2003; Tunnicliff and Malatynska, 2003) as well as converging reports of normalization of occipital cortex GABA concentrations after targeted therapy (Sanacora et al., 2002, 2003), further support the notion that GABA function highly contributes to the pathophysiology and treatment of depression. Along the same lines, increased PSC of the visual networks, as revealed by our study, putatively reflects the twofold mechanism underlying the overall changes in cortical excitability: (1) deficits in inhibitory processes governed by reduced GABA concentration, accompanied by (2) excessive excitatory stimulation due to increased glutamate concentration.

Studies in FC analysis have established a link between activity of ventral tegmental area (VTA) of the SN with occipital regions. Seed-based analysis shows an anti-correlated relation between VTA and the visual areas (Tomasi and Volkow, 2014; Zhang et al., 2016). Interpreted as inhibitory influence of SN on these areas, SN malfunctioning could relate to less inhibition.

## Limitations

In this study, we applied the SC as an informative summary measure reflecting spectral properties of RSNs. However, it is important to note that the SC is an averaged representative of the center of gravity of a network’s full power spectrum over time. Recent studies have, however, shown that the frequency content of RSN fluctuations is dynamic in time (Yaesoubi et al., 2015, 2017). Another concern that could be expressed over our findings, is the undermined neural information content of high-frequency fMRI signal. It has been suggested that the higher band BOLD signal is primarily driven by confounding factors, such as physiological noise, and that information specific to RSNs is limited to lowest frequency range of 0.01–0.1 Hz (Cordes et al., 2001). However, there is convincing evidence that this assumption might be wrong, as studies report meaningful neural content and resting-state connectivity patterns at frequencies up to 0.25 Hz or even 0.75 Hz (Niazy et al., 2011; Boubela et al., 2013; Lewis et al., 2016). Furthermore, here we operate on ICA-derived time courses, which are likely to contain fewer artifacts than the raw fMRI time courses. Moreover, the caveat that relates to potential non-neural effects in the low-frequency fMRI signal (<0.15 Hz) should also be considered. In addition to scanner drifts, low frequencies can also reflect aliased high-frequency cardiac pulsations (Lowe et al., 1998; Bhattacharyya and Lowe, 2004) and slow physiological changes such as end-tidal CO_2_ fluctuations (Wise et al., 2004). The effects of cardiac pulsations on group differences in SC were minimal, since the pulsation rates did not differ between groups. However, CO_2_ effects can be quite strong and are unlikely to be separated by ICA, as they constitute a global confound distributed across all ICs in the brain.

## Conclusion

In this work, we propose a new aggregate measure–the Spectral Centroid–which represents the “center of gravity” of the full power spectrum of individual RSN time courses. Based on a high-quality and large sample dataset of the Human Connectome Project, we show that there is a highly organized grading of SC across RSNs. This indicates a characteristic balance between specific frequencies involved in the power spectrum of each RSN. The occurrence of grading of SC across RSNs was replicated in an independent dataset, which further supports the validity of the proposed approach. Moreover, SC was shown to be a measure sensitive to power changes in BOLD network fluctuations in disease. In MDD, significantly increased SC was observed in the SN–a system well-known to be implicated in depression. Following the preliminary indication of altered spectral properties of SN in depression, we selectively investigated the spectral power of BOLD network fluctuations within distinct frequency bands. Compared to HCs, increased contributions of high-frequencies and reduced contributions of low-frequencies to the BOLD signal of SN were revealed in depression. In summary, SC is a compact and reliable measure that allows to determine characteristics of power distribution of BOLD network fluctuations and spot key changes in disease.

## Ethics Statement

This study was carried out in accordance with the recommendations of Human Research Committee guidelines of the Klinikum rechts der Isar, Technische Universität München with written informed consent from all subjects. All subjects gave written informed consent in accordance with the Declaration of Helsinki. The protocol was approved by the Human Research Committee of the Klinikum rechts der Isar, Technische Universität München.

## Author Contributions

AR, CS, and AW conceived the research. AR and CM preprocessed the data. AR and AW analyzed the data and prepared the figures. AR, CC, CS, and AW interpreted the results. AR drafted the manuscript. AR, CC, CM, CS, and AW edited and revised the manuscript. AR, CC, SG, CM, CS, and AW approved the final version of the manuscript.

## Conflict of Interest Statement

The authors declare that the research was conducted in the absence of any commercial or financial relationships that could be construed as a potential conflict of interest.

## Footnotes

1 https://www.humanconnectome.org

2 https://www.humanconnectome.org/study/hcp-young-adult/document/900-subjects-data-release

3 https://fsl.fmrib.ox.ac.uk/fsl/fslwiki/MELODIC

4 https://db.humanconnectome.org

5 http://mialab.mrn.org/data/hcp/RSN_HC_unthresholded_tmaps.nii

6 http://www.fil.ion.ucl.ac.uk/spm/

7 http://restfmri.net/forum/REST

8 https://www.nitrc.org/projects/pestica/

9 http://icatb.sourceforge.net

10 http://research.ics.aalto.fi/ica/icasso
