# Supplementary material for: Grading of Frequency Spectral Centroid Across Resting-State Networks
Source: Front Hum Neurosci. 2018 Oct 26;12:436. doi: 10.3389/fnhum.2018.00436 (PMC6213969; doi:10.3389/fnhum.2018.00436)
Supplement: Supplementary file 1 [file Data_Sheet_1.docx]

***Supplementary Material***

**Grading of frequency spectral centroid across resting-state networks**

**Anja Ries^1,2^, Catie Chang^3^, Sarah Glim^1,2,4^, Chun Meng^1,2,5^, Christian Sorg^1,2,6^, Afra Wohlschläger^1,2 *^**

^1^ Department of Neuroradiology, Technical University of Munich TUM, Munich, Germany

^2^ TUM-Neuroimaging Center, Technical University of Munich TUM, Munich, Germany

^3^ Advanced MRI Section, Laboratory of Functional and Molecular Imaging, National Institute of Neurological Disorders and Stroke, National Institutes of Health, Bethesda, Maryland, USA.

^4^ Graduate School of Systemic Neurosciences, LMU Munich, Planegg-Martinsried, Germany

^5^ Department of Psychiatry, University of Cambridge, Cambridge, United Kingdom

^6^ Department of Psychiatry, Technical University of Munich TUM, Munich, Germany

**Table of Contents**

1. ***Resting-state network (RSN) identification across study sites*** *2*

*Table 1. Correspondence between RSNs across study sites 2*

1. ***Dataset 1: Human Connectome Project*** *4*

*Table 2. RSN spectral centroid and connectivity*  *4*

*Correlation between spectral centroid and RSN size 5*

1. ***Dataset 2: Healthy controls and MDD patients*** *5*

*MDD patient description 5*

*Figure 1. Spatial maps of RSNs 6*

*Table 3. RSN connectivity 7*

*Table 4. Summary table of RSN outcome measures 9*

*Figure 2. Seed-based FC of salience network 10*

1. ***References*** *11*
2. **Resting-state network identification across study sites.**

Independent component analysis (ICA) was separately performed on data from both studies: (i) Dataset 1: the Human Connectome Project (Van Essen et al., 2013) (HCP; parcellation scenario with 50 ICs, ICA data freely available for download at https://db.humanconnectome.org) and (ii) Dataset 2: our study involving 25 healthy controls (HC) and 25 MDD patients (parcellation scenario with 75 independent components; ICs). For an automated identification of networks-of-interest from components, multiple spatial regression analyses on ICs were applied separately for both datasets using established and online available templates from Allen et al. (2011), reflecting canonical resting-state networks (RSNs; http://mialab.mrn.org/data/hcp/RSN_HC_unthresholded_tmaps.nii). Components of highest correlation coefficient (threshold at r > 0.2) with the templates were selected for further analysis. This resulted in selecting 24 RSNs for the Dataset 1, as well as 24 RSNs for Dataset 2. The names of selected RSNs, and their correspondence between both studies is presented in Supplementary Table 1.

**Supplementary Table 1. Correspondence between RSNs across study sites.** Correspondence between established resting-state network (RSN) templates provided by Allen et al. (2011) and RSN identified from the Human Connectome Project data (Dataset 1), as well as from our data of 25 HC & 25 MDD patients (Dataset 2). The table represents the IC number from Allen et al. (2011), the corresponding RSN name, the abbreviated RSN name used for the purposes of our work, and an indication whether IC from our Dataset 1 and Dataset 2 could be attributed to given RSN template. Abbreviations: DMN, default-mode network; CEN, central executive network.

| **Allen et al. (2011)** | |  | **IC attributed to RSN template?** | |
| --- | --- | --- | --- | --- |
| **IC** | **RSN name** | **RSN name abbreviated** | **Dataset 1** | **Dataset 2** |
| 21 | Basal Ganglia | BG | yes | yes |
| 25 | DMN anterior-medial | DMN_antmed | yes | yes |
| 68 | DMN anterior | DMN_ant | yes | yes |
| 53 | DMN posterior-lateral | DMN_postlat | yes | yes |
| 50 | DMN posterior | DMN_post | yes | yes |
| 34 | Attention left | ATT_L | yes | yes |
| 60 | Attention right | ATT_R | yes | yes |
| 72 | Attention | ATT | yes | yes |
| 71 | Attention Dorsal CEN | CEN | yes | yes |
| 55 | Salience Network | SN | yes | yes |
| 52 | Attention left | - | no | no |
| 17 | Auditory | AUD | yes | yes |
| 47 | Frontal | FRONT_1 | yes | yes |
| 49 | Frontal | FRONT_2 | yes | yes |
| 20 | Frontal | FRONT_3 | yes | no |
| 42 | Frontal | - | no | no |
| 38 | Sensorimotor | SM_1 | yes | yes |
| 7 | Sensorimotor | SM_2 | yes | yes |
| 56 | Sensorimotor | SM_3 | no | yes |
| 23 | Sensorimotor left | SM_L | yes | yes |
| 24 | Sensorimotor right | SM_R | yes | yes |
| 29 | Sensorimotor | SM_4 | yes | yes |
| 39 | Visual | VIS_1 | yes | yes |
| 46 | Visual | VIS_2 | yes | yes |
| 59 | Visual | VIS_3 | yes | yes |
| 64 | Visual | VIS_4 | yes | yes |
| 67 | Visual | VIS_5 | no | yes |
| 48 | Visual | VIS_6 | yes | no |

1. **Dataset 1: Human Connectome Project**

24 RSNs were identified from the ICs of Dataset 1. The spatial maps of RSNs are displayed in Figure 1 of the main material. Main activation sites representing the positive functional connectivity (FC) of a network are listed in Supplementary Table 2 (brain regions were identified from spatial maps thresholded at z > 15 using the Anatomy toolbox in SPM 12 (Eickhoff et al., 2005, 2006, 2007). Spectral centroid (SC) values were calculated on time courses of individual RSNs. For each of the 24 RSNs the mean values of SC are displayed in Supplementary Table 2.

***Supplementary Table 2. Dataset 1: Resting-state networks: spectral centroid and connectivity.*** *For each RSN the table represents the value of the corresponding spectral centroid (SC) as well as brain regions constituting the positive functional connectivity (FC) pattern within the network. RSNs are ordered according to their increasing SC values. Abbreviations: ACC, Anterior Cingulate Cortex; IFG, Inferior Frontal Gyrus; IPL, Inferior Parietal Lobule; MCC, Midcingulate Cortex.*

| **Dataset 1: Human Connectome Project** | | |
| --- | --- | --- |
| **RSN** | **SC [Hz]** | **positive FC** |
| DMN_postlat | 0.083 | Lingual Gyrus, Cuneus, Calcarine Gyrus, Precuneus, IPL, Angular Gyrus, MCC, Superior Frontal Gyrus, Fusiform Gyrus, |
| SM_1 | 0.085 | IPL, Supramarginal gyrus, IFG |
| ATT_L | 0.089 | Superior Frontal Gyrus, Middle Frontal Gyrus, Angular Gyrus, IPL, Middle Orbital Gyrus, IFG, MCC |
| ATT_R | 0.090 | Superior Parietal Lobule, IPL, Middle Frontal Gyrus, IFG, Superior Frontal Gyrus |
| VIS_4 | 0.090 | V2, Calcarine Gyrus, Lingual Gyrus, Cuneus, Superior Occipital Gyrus, Dorsal and Ventral Extrastriate Cortex (V3) |
| DMN_post | 0.091 | PCC, Cuneus, Precuneus, IPL |
| VIS_6 | 0.091 | Fusiform Gyrus, Lingual Gyrus, Middle Occipital Cortex, Lateral Occipital Cortex, Middle Temporal Gyrus, area V5/MT, |
| VIS_3 | 0.093 | Cuneus, Superior Occipital Gyrus, Lingual Gyrus |
| SN | 0.098 | ACC, Insula, MCC, Posterior-Medial-Frontal |
| FRONT_3 | 0.100 | IFG, Posterior-Medial-Frontal |
| SM_2 | 0.101 | Postcentral Gyrus |
| FRONT_1 | 0.104 | Insula, IFG, Middle Frontal Gyrus, Superior Medial Gyrus, Caudate Nucleus |
| VIS_2 | 0.104 | Calcarine Gyrus, V1 |
| ATT | 0.112 | Superior Parietal Lobule, Posterior-Medial-Frontal, MCC, Supramarginal Gyrus, |
| CEN | 0.113 | IFG, Middle Temporal Gyrus, Posterior-Medial-Frontal, Supramarginal Gyrus |
| DMN_ant | 0.116 | Middle Frontal Gyrus, Superior Frontal Gyrus, MCC |
| VIS_1 | 0.118 | Middle Temporal Gyrus, area V5/MT |
| DMN_antmed | 0.119 | Superior Medial Gyrus, Insula, IFG, MCC |
| FRONT_2 | 0.121 | Middle Frontal Gyrus, ACC |
| AUD | 0.125 | Superior Temporal Gyrus |
| SM_L | 0.125 | Precentral Gyrus |
| SM_4 | 0.131 | Paracentral Lobule |
| SM_R | 0.134 | Precentral Gyrus |
| BG | 0.215 | Putamen, Caudate Nucleus |

***Spectral Centroid & network size.*** We investigated the relation between RSN size and its corresponding SC value. Pearson’s correlation was computed between RSN size and SC value. A significant negative correlation was revealed (r = -0.54, p = 0.007). To rule out any differences in spectral properties related to the size of a network, we performed a regression analysis on the SC values and the sizes of RSNs. Regression residuals were taken to represent the SC value corrected for the network size. Friedman’s test with RSN as main factor was performed on the SC values corrected for network size. Results of the Friedman’s test revealed an intact grading of SC after controlling for network size χ^2^(23) = 10666.7, p < 0.001). Thus, we conclude that although the SC value correlates with the size of a given network, the grading of SC occurs independently of it.

1. **Dataset 2: healthy controls & MDD patients**

***MDD patient description.*** Recurrent major depressive disorder (MDD) was the primary diagnosis for all patients. All patients met the criteria for a current major depressive episode (MDE), where the average episode length was 16.6 weeks (SD = 6.62), the average HAM-D score was 22.12 (SD = 2.47), and the average BDI score was 24.08 (SD = 6.31). The mean duration of MDD was 16.72 years (SD = 10.20), with a mean number of episodes of 5.56 (SD = 2.47). The average GAF-score was 49.80 (SD = 10.53). Fourteen MDD patients had psychiatric co-morbidities, including generalized anxiety disorder (n = 6), avoidant or dependent personality disorder (n = 5), and somatization disorder (n = 3). Exclusion criteria for the patients included psychotic symptoms, schizophrenia, schizoaffective disorder, bipolar disorder, and substance abuse. Additional exclusion criteria for both groups were pregnancy, neurological or severe internal systemic diseases, and general contraindications for MRI. One MDD patient was not undergoing psychotropic medication treatment by the time of MRI assessment. Seven patients were treated by antidepressant mono-therapy [three cases: citalopram 30 mg/d (mean dose); three cases: sertraline 200 mg/d; one case: mirtazapine 30 mg/d]; 12 patients by dual-therapy (five cases: citalopram 37.5 mg/d + mirtazapine 30 mg/d; two cases: citalopram 40 mg/d + venlafaxine 225 mg/d; one case: citalopram 30 mg/d + quetiapine 200 mg/d; one case: sertraline 200 mg/d + mirtazapine 30 mg/d; three cases: venlafaxine 225 mg/d + mirtazapine 30 mg/d); and five patients by triple-therapy (two cases: citalopram 30 mg/d + venlafaxine 187.5 mg/d + amisulprid 200 mg/d; two cases: citalopram 30 mg/d + mirtazapine 30 mg/d + quetiapine 200 mg/d; 1 case: venlafaxine 22 mg/d + mirtazapine 30 mg/d + quetiapine 200 mg/d). All healthy control (HC) individuals were free of any current or past neurological or psychiatric disorder or psychotropic medication.

***Resting-state networks****.* Group-level spatial ICA on resting-state fMRI data from Dataset 2 was used to identify 24 RSNs (following the framework from Allen et al., 2011). RSNs are displayed in Supplementary Figure 1 and the corresponding names and activation sites representing the positive FC are shown in Supplementary Table 3. For each RSNs, the mean values of SC, percent signal change (PSC) and spectral centroid corrected for PSC (SCcorr) are displayed in Supplementary Table 4, separately for healthy controls (HC) and MDD patients.


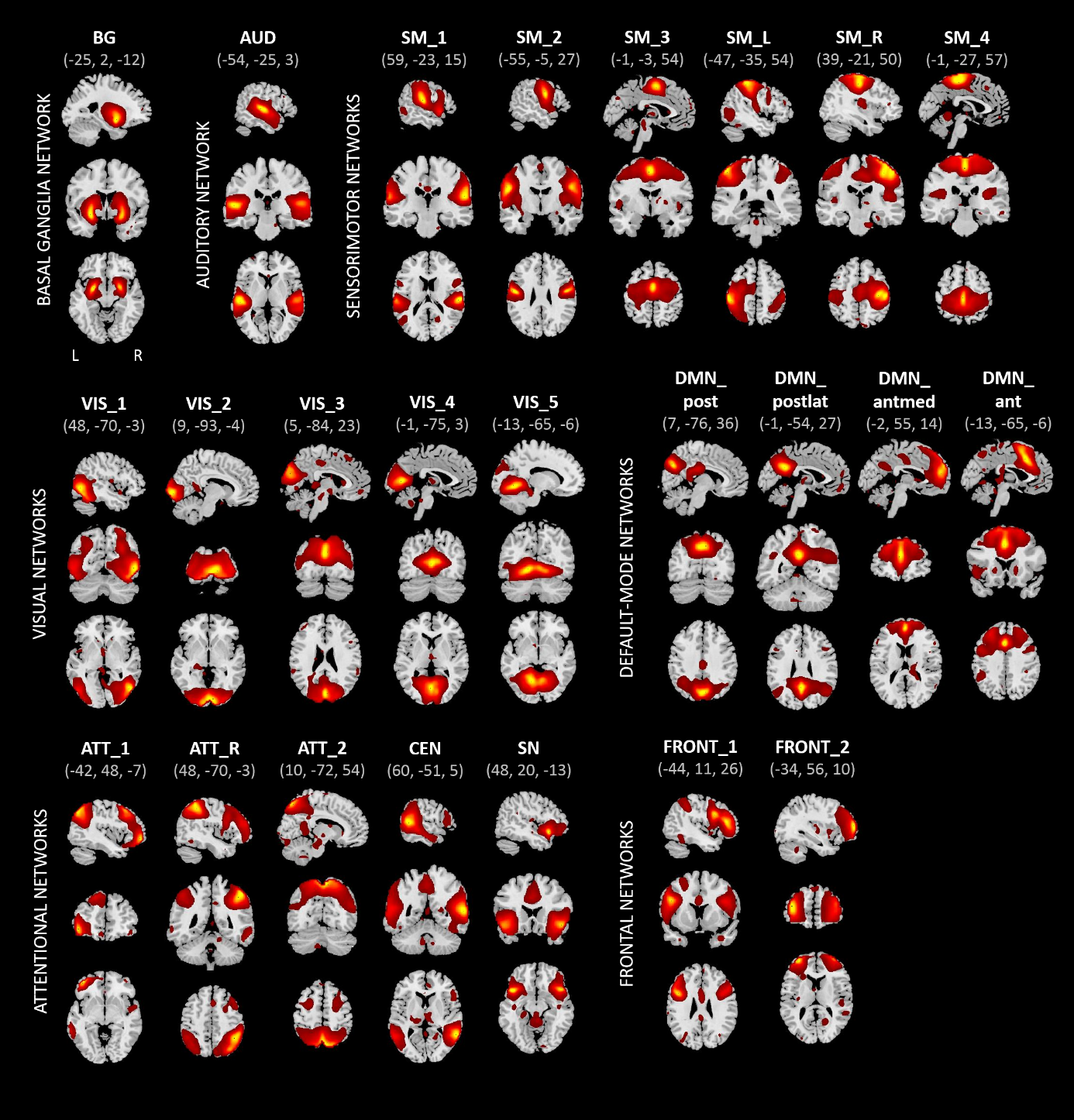
 **Supplementary Figure 1.** Dataset 2: Spatial maps of the 24 independent components identified as RSNs, obtained from spatial group-ICA. Spatial maps are plotted as z-scores, thresholded at z > 1, and displayed at the three most informative slices (MNI-space). RSNs are categorized into groups according to their anatomical and functional properties.

**Supplementary Table 3. Dataset 2: Resting-state network connectivity.** The table displays resting-state network (RSN) names (corresponding to RSN templates from Allen et al., 2011) and brain regions constituting the positive functional connectivity (FC) pattern within the network.

| **Dataset 2: Healthy Controls & MDD Patients** | |
| --- | --- |
| **RSN** | **Positive FC** |
| BG | Parahippocampal Gyrus, Lentiform Nucleus, Subcallosal Gyrus, Lateral Ventricle, Inferior Frontal Gyrus, Claustrum, Caudate, Insula, Uncus, Medial Frontal Gyrus, Superior Temporal Gyrus |
| AUD | Superior Temporal Gyrus, Insula, Transverse Temporal Gyrus, Middle Temporal Gyrus, Precentral Gyrus, Postcentral Gyrus |
| SM_1 | Transverse Temporal Gyrus, Postcentral Gyrus, Superior Temporal Gyrus, Inferior Parietal Lobule, Insula, Precentral Gyrus |
| SM_2 | Precentral Gyrus, Postcentral Gyrus, Superior Temporal Gyrus, Inferior Frontal Gyrus, Transverse Temporal Gyrus, Insula, Middle Frontal Gyrus |
| SM_3 | Medial Frontal Gyrus, Superior Frontal Gyrus, Cingulate Gyrus, Middle Frontal Gyrus, Paracentral Lobule, Precentral Gyrus |
| SM_L | Postcentral Gyrus, Inferior Parietal Lobule, Precentral Gyrus, Middle Frontal Gyrus, Superior Parietal Lobule |
| SM_R | Postcentral Gyrus, Precentral Gyrus, Middle Frontal Gyrus, Inferior Parietal Lobule, Insula, Paracentral Lobule, Medial Frontal Gyrus, Cingulate Gyrus, Transverse Temporal Gyrus |
| SM_4 | Paracentral Lobule, Medial Frontal Gyrus, Precentral Gyrus, Postcentral Gyrus, Superior Frontal Gyrus, Precuneus, Superior Parietal Lobule |
| VIS_1 | Fusiform Gyrus, Middle Occipital Gyrus, Inferior Temporal Gyrus, Inferior Occipital Gyrus, Middle Temporal Gyrus, Cuneus, Superior Occipital Gyrus, Precuneus, Lingual Gyrus, Cerebellum |
| VIS_2 | Lingual Gyrus, Cuneus, Inferior Occipital Gyrus, Middle Occipital Gyrus, Fusiform Gyrus |
| VIS_3 | Precuneus, Cuneus, Middle Occipital Gyrus, Posterior Cingulate |
| VIS_4 | Lingual Gyrus, Cuneus, Posterior Cingulate, Precuneus, Cerebellum, Middle Occipital Gyrus |
| VIS_5 | Cerebellum, Lingual Gyrus, Fusiform Gyrus, Parahippocampal Gyrus, Middle Occipital Gyrus, Cuneus, Posterior Cingulate |
| DMN_post | Cingulate Gyrus, Precuneus, Posterior Cingulate, Cuneus, Inferior Parietal Lobule, Supramarginal Gyrus |
| DMN_postlat | Cingulate Gyrus, Posterior Cingulate, Precuneus, Cuneus, Middle Temporal Gyrus, Superior Temporal Gyrus, Angular Gyrus, Supramarginal Gyrus |
| DMN_antmed | Medial Frontal Gyrus, Superior Frontal Gyrus, Anterior Cingulate, Middle Frontal Gyrus, Cerebellum |
| DMN_ant | Cingulate Gyrus, Superior Frontal Gyrus, Medial Frontal Gyrus, Anterior Cingulate, Middle Frontal Gyrus |
| ATT_L | Angular Gyrus, Inferior Parietal Lobule, Superior Temporal Gyrus, Middle Frontal Gyrus, Supramarginal Gyrus, Precuneus, Middle Temporal Gyrus, Precentral Gyrus, Superior Parietal Lobule, Inferior, Medial Frontal Gyrus, Superior Frontal Gyrus, Inferior Frontal Gyrus, Cerebellum |
| ATT_R | Inferior Parietal Lobule, Precuneus, Superior Parietal Lobule, Angular Gyrus, Supramarginal Gyrus, Middle Frontal Gyrus, Inferior Frontal Gyrus, Postcentral Gyrus, Superior Frontal Gyrus, Cerebellum |
| ATT | Precuneus, Superior Parietal Lobule, Postcentral Gyrus, Angular Gyrus, Superior Occipital Gyrus |
| CEN | Superior Temporal Gyrus, Middle Temporal Gyrus, Supramarginal Gyrus, Inferior Parietal Lobule, Precuneus, Inferior Temporal Gyrus |
| SN | Inferior Frontal Gyrus, Superior Temporal Gyrus, Insula, Cingulate Gyrus, Anterior Cingulate, Medial Frontal Gyrus |
| FRONT_1 | Inferior Frontal Gyrus, Precentral Gyrus, Middle Frontal Gyrus, Inferior Parietal Lobule, Supramarginal Gyrus |
| FRONT_2 | Middle Frontal Gyrus, Superior Frontal Gyrus, Inferior Frontal Gyrus |

**Supplementary Table 4. Dataset 2: Summary table describing RSN outcome measures**. For each resting-state network (RSN, column A) the table represents the values of the spectral centroid (SC, column B), the SC corrected for percent signal change (PSC; SCcorr, column C), and PSC (column D); separately for healthy controls (HC) and MDD patients. RSNs are ordered according to the increasing SC magnitude in HC, the colour scale represents the magnitude range where green = low, yellow = middle, red = high.

| A | B | | C | | D | |
| --- | --- | --- | --- | --- | --- | --- |
|  | **SC [Hz]** | | **SCcorr** | | **PSC [%]** | |
| **RSN** | HC | MDD | HC | MDD | HC | MDD |
| DMN_postlat | 0.052 | 0.055 | -0.011 | -0.011 | 0.47 | 0.46 |
| Att_L | 0.054 | 0.054 | -0.017 | -0.017 | 0.32 | 0.34 |
| SM_1 | 0.058 | 0.055 | -0.008 | -0.015 | 0.40 | 0.38 |
| Att | 0.059 | 0.063 | -0.010 | -0.008 | 0.36 | 0.36 |
| Att_R | 0.060 | 0.059 | -0.010 | -0.011 | 0.35 | 0.36 |
| DMN_antmed | 0.061 | 0.066 | -0.007 | -0.004 | 0.38 | 0.38 |
| SM_2 | 0.063 | 0.068 | -0.006 | -0.003 | 0.35 | 0.33 |
| DMN_post | 0.065 | 0.064 | -0.004 | -0.006 | 0.35 | 0.35 |
| FRONT_1 | 0.066 | 0.063 | -0.005 | -0.009 | 0.33 | 0.32 |
| CEN | 0.067 | 0.075 | -0.003 | 0.003 | 0.34 | 0.31 |
| SM_L | 0.068 | 0.068 | -0.004 | -0.005 | 0.30 | 0.30 |
| SM_4 | 0.069 | 0.071 | -0.005 | -0.003 | 0.29 | 0.28 |
| VIS_3 | 0.071 | 0.069 | 0.001 | 0.001 | 0.35 | 0.44 |
| VIS_4 | 0.073 | 0.066 | 0.003 | -0.002 | 0.33 | 0.45 |
| VIS_2 | 0.075 | 0.061 | 0.006 | -0.005 | 0.37 | 0.47 |
| SM_R | 0.075 | 0.075 | 0.004 | 0.002 | 0.33 | 0.30 |
| DMN_ant | 0.076 | 0.079 | 0.003 | 0.006 | 0.29 | 0.30 |
| FRONT_2 | 0.076 | 0.078 | 0.004 | 0.006 | 0.30 | 0.32 |
| VIS_1 | 0.079 | 0.073 | 0.007 | 0.002 | 0.31 | 0.34 |
| SN | 0.080 | 0.095 | 0.009 | 0.023 | 0.32 | 0.31 |
| BG | 0.081 | 0.087 | 0.011 | 0.013 | 0.35 | 0.28 |
| VIS_5 | 0.081 | 0.077 | 0.007 | 0.005 | 0.27 | 0.33 |
| AUD | 0.083 | 0.086 | 0.015 | 0.015 | 0.38 | 0.35 |
| SM_3 | 0.091 | 0.093 | 0.020 | 0.021 | 0.32 | 0.32 |

***Seed-based FC of salience network.*** The salience network (SN) was chosen as a ROI in the seed-based FC analysis. The averaged time courses of the SN, obtained via ICA, were used as regressors in a 1^st^ level GLM analysis to reveal the FC of the SN towards the whole brain in each participant. Seed-based FC was computed at the full power spectrum. Subsequently, individual connectivity maps for the SN were subjected to a one-sample t-test, separately for MDD patients and HC (p < 0.05, FWE-corrected). To test for group differences in whole brain seed-based FC of the SN, we applied a two-sampled t-test (p cluster-level corrected < 0.05, on underlying voxel-level correction of p < 0.001, with voxels restricted to gray matter).

In healthy controls, the FC pattern of the SN spanned bilaterally across the insula, inferior frontal gyrus, superior temporal gyrus, thalamus and cuneus (see Supplementary Figure 2A). There were significant alterations in the FC of the SN in MDD patients. Specifically, we observed decreased FC towards the bilateral middle frontal gyrus and ACC, and the right cuneus in patients (see Supplementary Figure 2B). The regions with aberrant FC to the SN could be attributed to the visual networks (VIS_1, VIS_2, VIS_4), frontal networks (FRONT_1, FRONT_2) and the anterior and anterior-medial DMN. Moreover, the anterior DMN and the visual network (VIS_4)—two networks of reduced FC towards SN also yielded a significant correlation and a trend, respectively, between the corresponding SCcorr value and the symptom severity (assessed through BDI scores).

We also performed frequency-resolved analysis of SN FC. However, the power within the band-limited regimes (i.e., at 10 distinct frequency bands) was too low to provide significant results. Band specific group differences only appear on a very liberal significance level (p < 0.05 uncorrected).


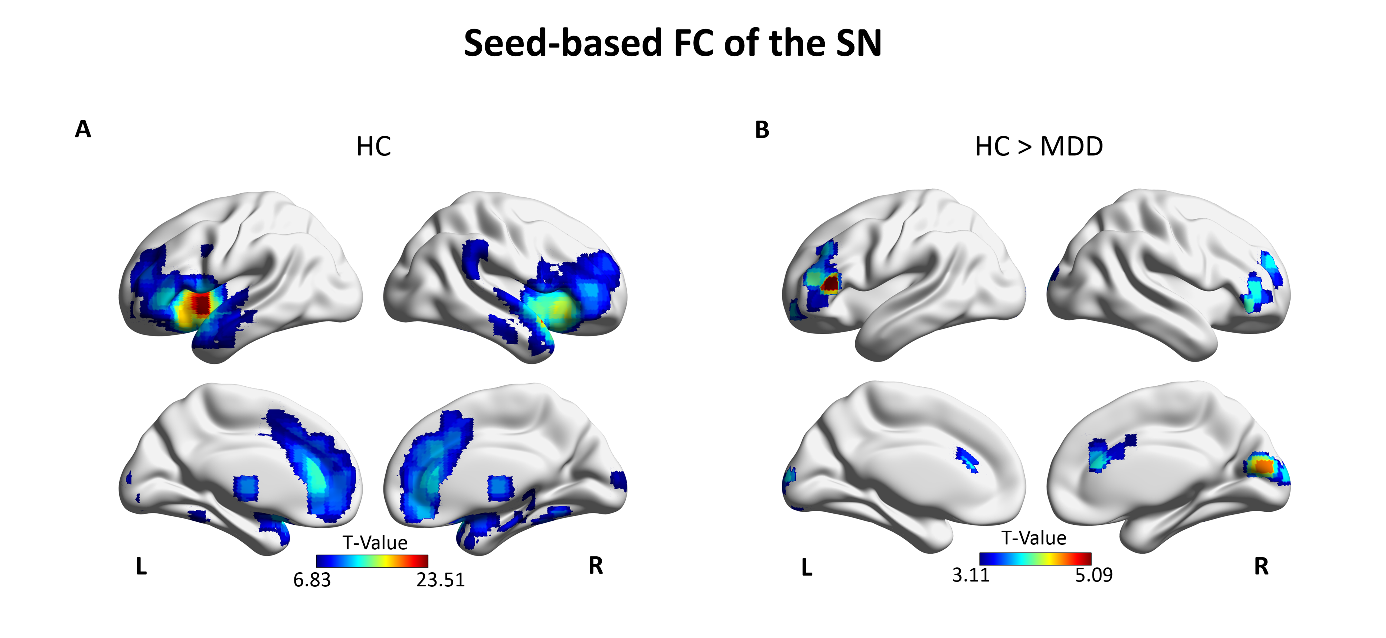


**Supplementary Figure 2** Seed-based functional connectivity (FC) of the salience network (SN). **(A)** Whole-brain seed-based FC of the SN in healthy controls (HC; p < 0.05, FWE-corrected) and **(B)** group differences in whole-brain seed-based FC of the SN, representing regions of decreased FC towards the SN in MDD patients as compared to HC (p cluster-level corrected < 0.05, on underlying voxel-level correction of p < 0.001).

**References**

Allen, E. A., Erhardt, E. B., Damaraju, E., Gruner, W., Segall, J. M., Silva, R. F., et al. (2011). A baseline for the multivariate comparison of resting-state networks. *Front. Syst. Neurosci.* 5, 2. doi:10.3389/fnsys.2011.00002.

Eickhoff, S. B., Heim, S., Zilles, K., and Amunts, K. (2006). Testing anatomically specified hypotheses in functional imaging using cytoarchitectonic maps. *Neuroimage* 32, 570–582. doi:10.1016/j.neuroimage.2006.04.204.

Eickhoff, S. B., Paus, T., Caspers, S., Grosbras, M.-H., Evans, A. C., Zilles, K., et al. (2007). Assignment of functional activations to probabilistic cytoarchitectonic areas revisited. *Neuroimage* 36, 511–521. doi:10.1016/j.neuroimage.2007.03.060.

Eickhoff, S. B., Stephan, K. E., Mohlberg, H., Grefkes, C., Fink, G. R., Amunts, K., et al. (2005). A new SPM toolbox for combining probabilistic cytoarchitectonic maps and functional imaging data. *Neuroimage* 25, 1325–1335. doi:10.1016/j.neuroimage.2004.12.034.

Van Essen, D. C., Smith, S. M., Barch, D. M., Behrens, T. E. J., Yacoub, E., Ugurbil, K., WU-Minn HCP Consortium (2013): The WU-Minn Human Connectome Project: An overview. Neuroimage 80:62–79.
